# TRAUMA-INFORMED CARE IN LONG-TERM CARE SETTINGS

**DOI:** 10.1093/geroni/igae098.1552

**Published:** 2024-12-31

**Authors:** Kelly O’Malley, Jennifer Sullivan, Whitney Mills, Jane “Jenny” Driver, Jennifer Moye

**Affiliations:** VA Boston Healthcare System, Boston, Massachusetts, United States; VA Providence Healthcare System, Providence, Rhode Island, United States; VA Providence Health Care System, VA Providence Health Care System, Rhode Island, United States; VA Boston Healthcare System, Brockton, Massachusetts, United States; VA Boston Healthcare System, Boston, Massachusetts, United States

## Abstract

By older adulthood, most older adults will have been exposed to at least one potentially traumatic event. Older veterans are likely to experience three traumatic events during their lifetime, with 93% reporting exposure to at least one event. The prevalence of posttraumatic stress disorder (PTSD) among older adults is lower. Nevertheless, the long-lasting psychological effects of trauma can manifest in later life, exacerbated by the normative experiences of aging (e.g., medical illness, loss of loved ones, retirement) and encounters with medical settings. Receiving care in skilled nursing settings, may trigger traumatic memories or exacerbate PTSD of symptoms. As the population ages, more individuals will receive care in long-term care environments, leading to increased risk of worsening PTSD and re-engagement with past traumatic memories and experiences. Staff in long-term care facilities may not have skills or knowledge needed to address symptoms or reduce re-traumatization (i.e., utilize a trauma-informed care approach). Trauma-informed care is an approach that utilizes knowledge of the effects of trauma to meet survivors’ healthcare needs, through collaborative care and recognition of patient strengths in managing their health. This approach is mandated in skilled nursing facilities; however, no models of trauma-informed care practice in long-term care exist. This symposium will review the effects trauma and PTSD in later life, the effects of medical settings on PTSD, and provide a framework for implementing trauma-informed care in long-term care settings.

